# Craving and Drug Reward: A Comparison of Celecoxib and Ibuprofen in Detoxifying Opiate Addicts 

**Published:** 2017-10

**Authors:** Sara Jafari, Sana Khajehpour, Emran Mohammad Razzaghi, Kazem Heidari, Mehdi Soleimani, Padideh Ghaeli

**Affiliations:** 1 Department of Psychiatry, School of Medicine, Tehran University of Medical Sciences, Tehran, Iran.; 2 Research Center of Rational Use of Drugs, School of Pharmacy, Tehran University of Medical Sciences, Tehran, Iran.; 3 Iranian National Center for Addiction Studies, Tehran University of Medical Sciences, Tehran, Iran.; 4 Department of Epidemiology Investigations, School of Pharmacy, Tehran University of Medical Sciences, Tehran, Iran.; 5 Department of Clinical Pharmacy, Roozbeh Hospital, School of Pharmacy, Tehran University of Medical Sciences, Tehran, Iran.

**Keywords:** *Celecoxib*, *Desire for Drug Questionnaire*, *Ibuprofen*, *Opiate Craving*, *Pain*

## Abstract

**Objective:** Craving for substance abuse is a usual and complicated problem in patients, with opioid addiction who are in opioid detoxifying process. Craving has been added as one of the diagnostic criteria of substance use disorders in DSM-5. The present trial aimed at comparing the effects of celecoxib versus ibuprofen in reducing pain and decreasing the desire to use opiates in patients undergoing opiate detoxification (n = 32).

**Method:** A total of 32 patients (both inpatients and outpatients), who were undergoing opiate detoxification procedure and met the inclusion criteria entered this 4- week study. Participants who suffered from pain due to opiate withdrawal were randomized into 2 groups: Group 1 received celecoxib 200 milligrams once per day and group 2 received ibuprofen 400 milligrams 4 times per day. Self-reported Desire for Drug Questionnaire (DDQ) and 0-10 numeric pain scale were used at baseline and at the end of the study to evaluate changes in opiate craving and pain, respectively. Data analysis was done by SPSS-21 statistical software.

**Results:** In this study, 16 patients received celecoxib 200 milligrams once daily, and 16 received ibuprofen 400 milligrams 4 times daily. After 4 weeks of treatment with both ibuprofen and celecoxib, the results revealed that celecoxib and ibuprofen equally reduced the pain symptoms. After 4 weeks of treatment, with either ibuprofen or celecoxib, significant improvement was observed in decreasing the craving in the celecoxib group, but not in the ibuprofen group.

**Conclusion:** The study revealed a significant difference between the celecoxib and ibuprofen group in reducing craving in patients with opiate craving after 4 weeks of treatment. However there were no significant differences between these two groups in reducing pain.

Craving is very common in patients who undergo opiate detoxifying procedure. Although drug craving has been defined in numerous ways, it has generally been regarded as a compelling urge to use a substance (1). Craving is usually thought to arise from either the positive reinforcing (incentive) properties of the drug (2, 3), the negative-reinforcing properties related to Withdrawal effects (4-6), or from a combination of both positive and negative reinforcement processes (7). In the recent years, it has been found that opiates and psychomotor stimulants activate appetitive motivational systems of the brain, which generate a positive affective motivational state (8, 9).

Several mechanisms are involved in drug craving, some of which are as follow: Dopamine system (10), decision-making system (11), serotonin System(12), oxytocin System (13) and stress system (14).

Researchers have long posited a relationship between craving and addiction (15). Abstinent opiate abusers, who just completed a 30-day treatment program in a therapeutic community setting, experienced intense drug craving (16). There are 3 major features of addictive behavior. The first is drug craving by which we simply mean intensely ‘wanting’ drugs (17).The second major feature of addiction by which we mean why drug craving often can be reinstated long after the discontinuation of drug use. A third feature of drug addiction is that as drugs come to be ‘wanted’ more-and-more, they often come to be ‘liked’ less-and-less. For instance, as craving for drugs increases, the pleasure derived from drugs often decreases (18).

Non-steroidal anti-inflammatory drugs are inhibitors of the cyclooxygenase enzyme family, which catalyzes the metabolism of arachidonic acid to prostaglandins, prostacyclin, and thromboxane. The cyclooxygenase-1 isoform is constitutively expressed in most tissues. The cyclooxygenase-2 is usually specific to inflamed tissue (19).

Celecoxib is a COX-2 inhibitor that has been used in many psychiatric problems such as major depression by inhibiting prostaglandin E2, which may be raised in MDD (20) and reducing the signs of obsession and compulsions as an adjuvant therapy in obsessive-compulsive disorders (21).

In our practice, we noted that many psychiatrists believe that celecoxib causes more craving inhibitory effect when compared with other nonsteroidal anti-inflammatory drugs (NSAIDs)and when being used as an analgesic in patients who have craving during their opiate cessation procedure. The researches reflect the action on celecoxib on some parts of the brain involving craving (22). To our knowledge, there have not been any published data on the comparison between celecoxib and ibuprofen for their effects on craving in opiate cessation craving. Therefore, this 4-week study has been designed to compare the effects of celecoxib and ibuprofen on opiates cessation craving in patients with a diagnosis of addiction based on Diagnostic and Statistical Manual for Mental Disorders-Text Revision 5th Edition (DSM-V) criteria (23).

The present study tested the theory that celecoxib would differ from ibuprofen in measuring the desire to use opioids because of its positive effects on brain, and also it was expected to lower the pain in the 2 groups of the study (24).

In summary, this study aimed at testing whether the inhibition of craving effect, assessed terms of increased desire to use opioid for their rewarding effects, would be greater in the celecoxib group than the ibuprofen group, following administration of the 4-week therapy using both medicines.

## Materials and Methods

***Participants***

In this study, 32 opiate addicts, who met DSM-5 criteria for opioid use disorder, participated. Patients were under a detoxification protocol at addiction clinics in Tehran, Iran. They were randomly allocated into 2 groups and they received a 30-day celecoxib treatment or a 30-day ibuprofen treatment for their pain during their detoxifying period. The inclusion criteria included patients aged 18 and 65 who were admitted to Roozbeh hospital clinic and a private clinic to receive opioid cessation regimens.

The exclusion criteria included pregnancy and lactation, suffering from severe medical conditions (eg, cancer, cardiovascular and cerebrovascular diseases, thyroid diseases, and any gastrointestinal diseases), or neurological illnesses including epilepsy and Parkinsonism, having received NSAIDs within the week prior to the initiation of the study as well as having intellectual disability.

There were no significant differences in the demographic variables or drug history between the participants of the 2 groups. All participants were identical in environment and lifestyle during the trial; for instance, they were received similar diet and drug regimen and the same psychiatric counseling during their therapy. Moreover, they were asked not to change their usual lifestyle during the trial. The detoxifying procedure was done by standard psychiatric counselling of psychiatrists and psychologists. No other routine detoxifying medications (such as methadone) were used.

***Procedure***

Each patient participating in this study was assessed for pain and craving for opiates at baseline. The patients were assigned to receive either celecoxib or ibuprofen randomly for 4 weeks. Patients in celecoxib group received celecoxib capsule 200 milligrams orally once daily at 12 PM. Ibuprofen was administered in a soft gel form and in an equal dose of celecoxib, which is 1600 milligrams in 4 divided doses at 08:00 AM, 02:00 PM, 08:00 PM and 12:00 midnight (25). The dosages could be further increased to 200 mg 3 times daily for celecoxib and 400 milligrams 6 times daily for ibuprofen if needed clinically per decision of the psychiatrist in charge. Changes in pain and craving were obtained at baseline and Week 4 of the treatment, using Desire for Drug Questionnaire. The person in charge of assessing the patients’ pain and craving was not aware of the patients’ medication, however, each patient was aware of his treatment.

A psychiatry resident randomly assigned the patients to receive treatment with either celecoxib or ibuprofen under single-blind conditions and allocated them to parallel groups, which was determined by simple randomization. He kept the randomization list secure during the study.

After being provided with a full explanation of the protocol design, all patients provided written informed consent. This trial was approved by the Ethics Committee of the Faculty of Pharmacy at Tehran University of Medical Sciences and registered in the Iranian Registry of Clinical Trials (www.irct.ir; identifier: IRCT201609207202N11). All phases of this study were performed according to the declaration of Helsinki on ethical principles for medical research involving human subjects.

***Questionnaires and Measures***

The 14-item Desire for Drug Questionnaire (DDQ) is an effective tool for subjective assessment, showing how strong the desire is to use opiates (Cronbach’s alpha: 0.89, 0.84, and 0.37; test-retest reliability of r = 0.83, r = 0.82 and r = 0.74 for desire, negative reinforcement and control, respectively) (26). It is a self-report questionnaire, which contains 14 questions. The original DDQ includes 13 questions for 3 main craving components: desire and intention to drug use (Questions 1, 2, 4, 6, 10, 13, and 14), negative reinforcement (Questions 5, 9, 11, and 12), and control (Questions 3 and 8). We restored the question that had been excluded from the final questionnaire by Franken, et al. (2002) to increase the internal consistency of its respective component in our questionnaire (Question 7). Participants answered the questions on a seven-step Likert-scale answer sheet based on what they felt or thought at the moment. The items were rated as follow: (1) not at all; (2) mild; (3) mild to moderate; (4) moderate; (5) moderate to severe; (6) severe, and (7) approximately complete. Originally, the scale was 

this study to assess the intensity of the patients’ pain. It is a numeric scale, ranging from 0-10, with zero indicating no pain and 10 the worst possible pain (28).

Prior to the beginning of the study, sample size was calculated to detect a 20% difference in improvement between celecoxib and ibuprofen. Under the assumption of a significant level of 0.05 with a power of 0.80, a minimal sample size of 32 with 16 cases in each group was determined. Estimating a dropout rate of 20%, we decided to recruit 20 participants for each group.

***Data Analysis***

Data analysis was done by SPSS-21 statistical software. The changes in pain and craving were detected at Weeks 0 and 4 between the 2 groups. Statistical significance was set at p < 0.05.

## Results

*Demographic Characteristics*

Demographic characteristics of the patients in both groups are demonstrated in Table 1. In this study, 40 male patients were screened for the study, of whom 8 were excluded. Chart 1 displays the patient selection procedure during the trial. In this study, 32 patients completed the trial; 16 men were prescribed celecoxib (mean age in years ± standard deviation was 34.625 ± 8.18) and 16 were on ibuprofen (mean age in years ± standard deviation was 31.625 ± 7.841). Developed in English; however, in this trial the Persian translation was used (27).

The 0-10 numeric pain scale (Cronbach’s alpha of 0.89 and test-retest reliability of r = 0.73) was also used in

**Figure 1:**
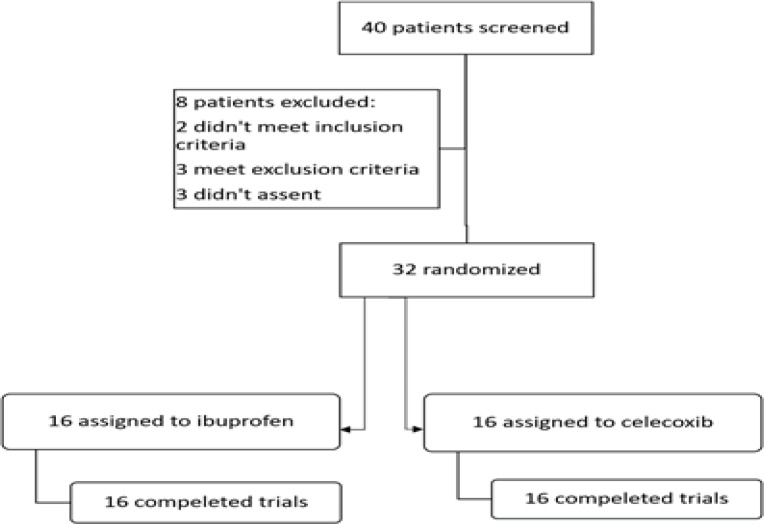
The procedure of allocating patients into two groups of celecoxib and ibuprofen

**Figure 2:**
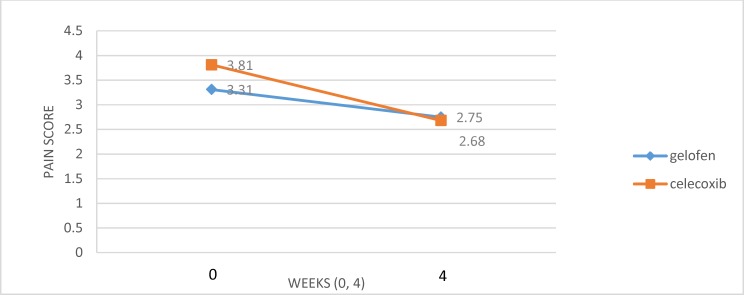
Effects of celecoxib vs ibuprofen on pain severity at weeks 0 and 4

**Figure 3:**
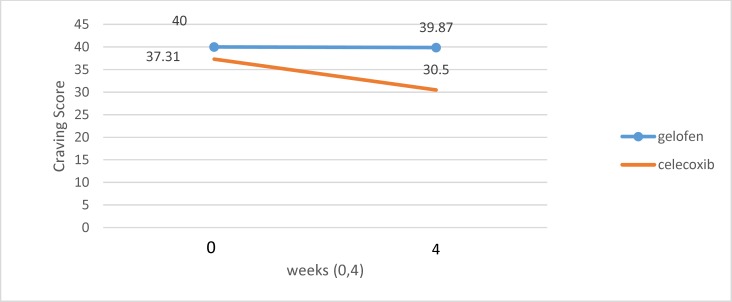
Effects of celecoxib vs ibuprofen on craving (DDQ) severity at weeks 0 and 4

**Table 1:** Baseline demographic and clinical charecteristics of patients

**Variable**	**Celecoxib**		**Ibuprofen**		**P (2-tailed)**
	No	%	No	%	
** Age**					0.29
Mean (SD)	16	34.625 (8.18)	16	31.62 (7.84)	
** Gender**					0.29
Male	16	100	16	100	
** Marital status**					0.48
Married	6	37.5	9	56.3	
Never married	10	62.5	7	43.8	
** Job status**					
Jobless	9	56.2	7	43.8	
Full time	7	43.8	9	56.3	
** Educational status**					
Primary school	2	12.5	5	31.25	
Guidance school	3	18.75	7	43.75	
High school	7	43.75	3	18.75	
Diploma or more	4	25	1	6.25	
** Kind of opioid**					
Heroin	9	56.3	14	87.5	
Methadone	5	31.2	0	0	
Opium	2	12.5	2	12.5	
** Pain severity**					0.218
Numeric pain scale		3.32 (1.27)		3.31 (0.95)	

1. Two-independent Sample t test                                      2. Standard Deviation

3. Two-independent Sample t test, χ2(n=32,1)=1.13            4. χ2 Square test, χ2(n=32,1)=0.5

**Table 2:** Means, Standard Deviation and ANCOVA results for the effects of celecoxib and ibuprofen on craving

		**Celecoxib**	**(n = 16)**		**Ibuprofen**	**(n = 16)**	
**Variable**	Pretest	Posttest	Paired	Pretest	Posttest	Paired	F _(1, 29)_
	M(SD)	M(SD)	t(15)	M(SD)	M(SD)	t(15)	
**Craving**	37.31(14.5)	30.5(9.33)	-3.43	40(13.53)	39.8(13.44)	0.126	12.944^1^

1. p value = 0.001

Participants who were assigned into the 2 groups did not differ in their gender, age, marital, job or educational status (Table 1). Analysis of Variance (ANOVA) was utilized to determine the equivalence of the treatment conditions on outcome measures at the pretest point. These results demonstrate the success of random assignment in producing equivalent groups on these variables at the pretest point. 

***Pain Severity***

At first for analyzing the scores the outlier test was passed. To analyze pain severity, independent samples Mann-Whitney U test, a non-parametric test, was used because the shapiro-wilk test showed a significant difference (p<0.05). The result of pain score is presented in Figure 2. Mean (SD) of pain severity in the celecoxib group was 3.32±1.27 at baseline and 2.75±1.61 at week 4, and it was 3.31±0.95 at baseline and 2.68±1.35 at week 4 in the ibuprofen group. Analysis of the pain using 0-10 numeric pain scale, showed significant difference between the pretest and posttest scores (p = 0.003) but no significant difference between the two treatments (p = 0.27) (Figure 2).

***Inhibitory Effects on Craving***

At the end of week 4, the normality, outlier and Levene test was passed so the analysis of covariance (ANCOVA) was used to determine the equivalences of the treatment conditions on outcome measures at the pretest point and the pre-hypothesis tests were done before analysis. The general linear model test also was not significantly different at the pretest (p=0.951, F_(1,28)_=0.004). The results did not indicate a significant difference at the pretest point for craving symptoms. So to compare the 2 groups of treatment, analysis of covariance (ANCOVA) was used with pretest score as the covariant. The ANCOVA results and the means and standard deviation of the outcome measures at the pretest and posttest are presented in Table 2. The results revealed an improvement in craving in the celecoxib group, but not in the ibuprofen group. According to the results, celecoxib is significantly different from ibuprofen in reducing opiate craving (F_(1, 29)_ = 12.94, p = 0.001) (29).

## Discussion

Animal experiments have illustrated that prostanoids mediate the results of morphine withdrawal pain in guinea pig ileum like contracture, so phospholipase A2, COX-1, COX-2, and 5-lipoxygenase inhibitors that block these mediators are expected to inhibit these effects. Dunbar et al. demonstrated that NSAIDs have been indicated to hyperpolarize neurons by increasing outward K ion conductance in opposition to the mechanism of PGE2 in enhancing withdrawal pain. Various pain models explained that the PGE2 secretion is a result of excitatory amino acid release, thus, NSAIDs are expected to block withdrawal pain, which is a positive effect during withdrawal (30).

Craving is considered as a risk factor for abusing drug after opiate cessation. The present study used a questionnaire to assess cravings and urges to use opiates. To the author’s knowledge, to date, no study has compared celecoxib with other drugs on opiates craving. Our study on opiates addict patients, who went under cessation procedure, showed no significant difference in decreasing the opiate craving after a 4-week treatment with celecoxib and ibuprofen. It should be also noted that the duration of this study was not long enough to show the chronic effects of the studied NSAIDs on opiate craving. 

Wise (1988) has proposed that the biological basis for drug ‘craving’ reflects 2 independent processes: one in which the primary objective is to alleviate an aversive state (eg, withdrawal), and the other in which appetitive motivational processes are involved (31).

Frolov’s trial shows that celecoxib as a COX-2 Inhibitor, can act in several parts of the brain and in those parts responsible for craving (22).

In another study, Dembo et al. found that celecoxib penetration into the CNS is indicated by the CSF plasma concentration ratio (32).

Ciceri et al. showed that celecoxib reduced the elevated PGE2 levels in the brain of kainate-treated rats and inhibited induced COX activity, demonstrating the ability of this compound to act on COX-2 in CNS (33).

Beiche et al. found that mRNA of both COX isoforms is expressed continuously in the spinal cord with COX-2 as the predominant isoform (34).

Celecoxib administration produces a decrease in the subjective desire to use opiates for their pleasant effects. There was no overall significant difference on this measure between the 2 groups at baseline so these results reflected no pre-existing differences prior to drug administration day. However, the pattern of results obtained revealed significant differences between the ibuprofen and celecoxib group on craving. However, no significant difference was obtained between the 2 groups of treatment on reducing pain.

The author suggests considering high risk patients such as patients who have GI diseases, cardiac diseases and etc., before starting celecoxib or ibuprofen for them. Furthermore, regular monitoring of the patients after initiating NSAIDs may prevent further complications. It is also important to consider any drug-drug interactions before initiating celecoxib or ibuprofen. In general, celecoxib or ibuprofen may be used for pain in opiate addict patients during their detoxifying procedure. Further studies are needed to assess the effects of chronic use of celecoxib and other NSAIDs in opiate addict patients.

## Conclusion

The study suggests that both celecoxib and ibuprofen improved pain related to opioid withdrawal in the studied patients. Furthermore, no significant difference was observed between the 2 groups in the assessment of alteration of pain perception. On the other hand, the trial revealed a significant difference between celecoxib and ibuprofen on craving for opiates, suggesting a positive evidence for consumption of celecoxib rather than ibuprofen for opiate craving in clinical settings. More randomized controlled trials with larger sample sizes and longer duration of follow- ups are needed to confirm the findings of this study.
